# Comparative Mapping of Seed Dormancy Loci Between Tropical and Temperate Ecotypes of Weedy Rice (*Oryza sativa* L.)

**DOI:** 10.1534/g3.117.040451

**Published:** 2017-06-06

**Authors:** Lihua Zhang, Jieqiong Lou, Michael E. Foley, Xing-You Gu

**Affiliations:** * Agronomy, Horticulture, and Plant Science Department, South Dakota State University, Brookings, South Dakota 57007; † Northern Crop Sciences Laboratory, United States Department of Agriculture-Agricultural Research Service, Fargo, North Dakota 58102

**Keywords:** seed dormancy, weed, comparative genomics, quantitative trait locus, segregation distortion

## Abstract

Genotypic variation at multiple loci for seed dormancy (SD) contributes to plant adaptation to diverse ecosystems. Weedy rice (*Oryza sativa*) was used as a model to address the similarity of SD genes between distinct ecotypes. A total of 12 quantitative trait loci (QTL) for SD were identified in one primary and two advanced backcross (BC) populations derived from a temperate ecotype of weedy rice (34.3°N Lat.). Nine (75%) of the 12 loci were mapped to the same positions as those identified from a tropical ecotype of weedy rice (7.1°N Lat.). The high similarity suggested that the majority of SD genes were conserved during the ecotype differentiation. These common loci are largely those collocated/linked with the awn, hull color, pericarp color, or plant height loci. Phenotypic correlations observed in the populations support the notion that indirect selections for the wild-type morphological characteristics, together with direct selections for germination time, were major factors influencing allelic distributions of SD genes across ecotypes. Indirect selections for crop-mimic traits (*e.g.*, plant height and flowering time) could also alter allelic frequencies for some SD genes in agroecosystems. In addition, 3 of the 12 loci were collocated with segregation distortion loci, indicating that some gametophyte development genes could also influence the genetic equilibria of SD loci in hybrid populations. The SD genes with a major effect on germination across ecotypes could be used as silencing targets to develop transgene mitigation (TM) strategies to reduce the risk of gene flow from genetically modified crops into weed/wild relatives.

Weeds are unwanted plants that have adapted to agroecosystems and compete with crop cultivars (Harlan 1965; Booth *et al.* 2003). Seed dormancy (SD) plays a critical role in the adaptation. Weed seeds, usually dormant upon maturation, may survive in the soil for months to years, depending on genotypes and environments. Presumably, the genotypic differentiation of SD in a species occurred at multiple loci during evolution before specific populations adapted to agroecosystems as weeds, given the relatively short history of domestication for major crops (<9000 yr; Chopra and Prakash 2002). Thus, it is important to know about the degree of similarity in SD genes between distinct ecotypes and factors influencing their genotypic/allelic frequencies. This information may help design new weed management strategies. We selected weedy rice as a model system to address the ecological genetic issues in this research.

Weedy rice refers to various forms of plants that belong to the *Oryza* genus and infest rice fields from tropical to temperate areas (Oka 1988; Delouche *et al.* 2007). The rice *Oryza sativa* was domesticated from the wild ancestor (*O. rufipogon* Griff.) and differentiated into the *indica* and *japonica* subspecies that are distributed across tropical/subtropical and temperate areas, respectively (Khush and Brar 2002). The origin of the conspecific weedy rice was associated with the domestication and subspeciation processes. For example, weedy rice populations can be *indica*- or *japonica*-like. The *indica*-like populations in tropical areas (tropical ecotypes) could originate from natural variants of the wild ancestor, or from hybrids between wild and cultivated rice. The *japonica*-like populations in temperate areas (temperate ecotypes) that are historically absent of wild rice may originate from old/extinct cultivars, or hybrids between *indica* and *japonica* cultivars (Suh *et al.* 1997; Tang and Morishima 1997). Despite the ecotype differentiation, weedy rice populations, particularly those adapted to an ecosystem for a long period, usually have strong SD and some other wild-type characteristics (Oka 1988; Delouche *et al.* 2007). The phenotypic similarity between distinct ecotypes could arise from the same or different sets of genes, depending on the relatedness of weed populations and the coevolutionary relationship between SD and the other adaptive or domestication-related traits in local ecosystems. We used a comparative mapping approach to infer the differentiation at QTL for SD (qSD) between temperate and tropical ecotypes of weedy rice.

Wild and weedy rice are divergent from cultivated rice in SD, as evaluated under controlled environment conditions (Veasey *et al.* 2004; Gu *et al.* 2005a). Several lines of wild (*O. rufipogan* or *O*. *nivara*)/weedy rice were crossed with cultivars to identify QTL associated with domestication-related traits, including SD or germination capacity (Cai and Morishima 2000; Thomson *et al.* 2003; Gu *et al.* 2005b; Lee *et al.* 2005; Li *et al.* 2006; Jing *et al.* 2008; Subudhi *et al.* 2012; Mispan *et al.* 2013). The number of reported SD QTL varied with mapping populations or environments (years) from a few to ∼20. Some of them remain to be confirmed because several factors in a distant cross, such as partial sterility or low seed set, segregation distortion, and seed shattering, could have a negative impact on the QTL analysis (Cai and Morishima 2000). In the previous research, we identified 10 SD QTL in a primary and advanced BC populations derived from a tropical ecotype of weedy rice (Gu *et al.* 2004; Ye *et al.* 2010). All of these 10 loci have been confirmed, and some of them have been cloned. The objectives of this research were to: (1) identify SD QTL for a temperate ecotype of weedy rice and (2) compare these loci with those mapped for the tropical ecotype to infer shared genetic and evolutionary mechanisms underlying the adaptive trait.

## Materials and Methods

### Parental lines and mapping populations

#### Two ecotypes of weedy rice:

The pure lines, LD and SS18-2, were selected from the previous research (Gu *et al.* 2005a) to represent the temperate and tropical ecotypes of weedy rice, respectively. LD was purified from “LüDao” (in Chinese), a population of volunteer rice historically present in the Lianyungang area (34.33–34.46°N Lat.) of East China (Jiang *et al.* 1985). This population was similar to some local landraces (*O. sativa* ssp. *japonica*) in plant type and seed (spikelet) morphology (black hull and red pericarp colors, long awn, and medium grain), but different from the old landraces in seed shattering and dormancy (Jiang *et al.* 1985). SS18-2 was purified from SS18, a population of weedy rice from the Songkla (7.18°N Lat.) area of Southern Thailand (Tang and Morishima 1997), and is similar to LD in seed morphology and dormancy (Supplemental Material, Table S1 in File S1). Despite the phenotypic similarity, there was no direct relationship in origin between these two geographically isolated weed populations. Based on diagnostic characteristics and isozyme markers, LD and SS18 were classified into the *japonica*- and *indica*-like groups of weedy rice, respectively (Tang and Morishima 1997).

#### Recurrent parent and BC populations:

EM93-1, an early maturation semidwarf *indica* line (Ye *et al.* 2013), was used as the female, recurrent parent to develop BC populations. The BC_1_F_1_ “EM93-1//EM93-1/LD” population, which had been previously evaluated for phenotypic correlations between seed-related traits (Gu *et al.* 2005a), was used to scan for QTL along the LD genome. In addition, two BC_1_F_1_ plants (#9 and 139), which were similar to EM93-1 in flowering time, were selected to develop the BC_2_F_1_ (9) “EM93-1/BC_1_F_1_ plant #9” and BC_2_F_1_ (139) “EM93-1/BC_1_F_1_ plant #139” populations. The BC_2_F_1_ (9) and (139) populations were used to confirm the detected QTL and to identify additional loci whose effects on germination may have been masked by some major genes segregating in the BC_1_F_1_ population (Ye *et al.* 2010).

### Plant cultivation, and seed harvesting and storage

The BC_1_F_1_ and BC_2_F_1_ populations were grown in greenhouses in different years. To capture all available genotypes in a mapping population, hybrid seeds were air-dried to break dormancy, germinated at 30° and 100% relative humidity for 5 d, and cultured with a nutrient solution (Yoshida *et al.* 1976) for 2 wk. Seedlings were transplanted into pots (28 cm diameter × 25 cm height), with one plant per pot, and filled with a mixture of clay soil and Sunshine #1 medium (Sun Gro Horticulture). Greenhouse temperatures were set at 29/21° for day/night, and the day-lengths were natural, except from the 6th to 8th wk when a 10-hr (8:00–18:00) short-day treatment was used to synchronize flowering. Plants were tagged for flowering dates when the first panicle in a plant emerged from the leaf sheath. Panicles were covered with white pollination bags at ∼10 d after flowering and the bags fixed to bamboo poles to prevent shattering due to brushing or shaking the plant. Seeds were harvested at 40 d after flowering, air-dried in the greenhouse for 3 d, and stored in a freezer (−20°) to maintain the status of dormancy developed on the plant (*i.e.*, primary dormancy).

### Phenotypic identifications for SD and morphologies

#### SD:

The primary dormancy was evaluated by germination percentage for both seeds and caryopses from the BC_1_F_1_ and for seeds from the BC_2_F_1_ populations. A “seed” in grass species usually refers to a dispersal unit, which consists of the seed component (embryo, endosperm, and testa) and covering (pericarp and hull, or lemma and palea) tissues, whereas a caryopsis is a hull-removed seed enclosed by the pericarp. To evaluate seed germination, after-ripening (AR) treatments were used to release part of the primary dormancy to better display genotypic variation on the percentage scale. Briefly, seeds from each plant were allocated into three or four sets and stored in a lab room (24–25°) for a series of 7 or 10 d intervals to obtain various degrees of partially AR samples. Caryopses were prepared by hand removal of the hull from non-AR seeds. About 50 seeds/caryopses were distributed in a 9 cm petri dish, which was lined with a filter paper and wetted with 8 ml deionized water. A germination experiment was replicated three times in an incubator set for 30°, 100% relative humidity, and dark conditions. Germinated seeds (radicle protrusion > 3 mm) were counted at day 7.

#### Awn:

The BC_2_F_1_ populations were evaluated for the morphological traits awn, hull color, and pericarp color, to confirm their correlations with SD in the BC_1_F_1_ “EM93-1//EM93-1/LD” population (Gu *et al.* 2005a). An awn is a needle-like appendage extended from the terminal end of a lemma and functions in aiding seed dispersal or movement into wet soil. The awn trait varies in length with plants, as well as with seeds on a panicle, in a segregating population. Thus, the trait was quantified by the mean awn length, and the percentage of seeds with an awn, in a random sample of >50 seeds from a BC_2_F_1_ plant.

#### Hull color:

This trait was measured with the ChromaMeter Minolta CR310, which transfers reflectance spectra into the L*, a*, and b* readings to quantify blackness, redness, and yellowness, respectively. The L* readings range from 0 to 100, with 0 and 100 indicating completely nonreflective (black) and perfectly reflective (white), respectively. The a* readings vary from −100 to 100, with negative and positive values indicating greenness and redness, respectively. The b* readings also vary from −100 to 100, with negative and positive values indicating blueness and yellowness, respectively. The reflectance spectra were measured using ∼100 seeds in a 6 cm petri dish on a dark background, and means of three independent readings for each of the spectra used for data analysis.

#### Pericarp color:

This trait was visually scored as red/brown (1) or white (0) for correlation analysis. This was partly because most BC_2_F_1_ plants had an insufficient amount of seeds to prepare caryopses for the reflection spectrum measurement after the replicated germination tests. In addition, the pigment trait is controlled by the gene *Rc* encoding a bHLH familiar transcription factor in rice (Sweeney *et al.* 2006; Furukawa *et al.* 2007). This regulatory gene is also one of the QTL for SD (*i.e.*, *qSD7-1*) and its functional alleles are present in tropical and temperate ecotypes of weedy “red” rice, including LD and SS18-2, to control maternal tissue-imposed dormancy (Gu *et al.* 2011).

### Marker genotyping and map construction

Fresh leaves were used to prepare genomic DNA samples for marker genotyping. More than 300 rice microsatellite markers from the 12 chromosomes (Chrs) of rice (McCouch *et al.* 2002), including all of those used to map the SS18-2 genome (Gu *et al.* 2004), were screened for polymorphism between EM93-1 and LD. Information on the markers (primer sequences, repeat motifs, and genomic positions) is available in the Gramene database (http://archive.gramene.org/markers/microsat/). DNA extraction, marker amplification by polymerase chain reaction (PCR), and PCR product display by electrophoresis in a 6% nondenatured polyacrylamide gel were performed using the previously described methods (Gu *et al.* 2004). Marker genotypes were scored using the AlphaEaseFC (Alpha Innotech) gel imaging system. Polymorphic markers with a size difference suitable to score were used to genotype the BC_1_F_1_s to develop a linkage map covering the weedy rice genome. Markers on the Chr or Chr segments heterozygous for the BC_1_F_1_ plants #9 and #139 were used to genotype the BC_2_F_1_ (9) and BC_2_F_1_ (139) populations, respectively, to develop partial linkage maps.

Linkage maps were constructed using MAPMAKER/EXP 3.0 (Lincoln *et al.* 1992). Map distances in centiMorgan (cM) were converted from recombination fractions using the Kosambi mapping function. Markers were grouped at the LOD score of 3.0 and the maximum distance of 50 cM (equivalent to 0.38 recombination fraction). Linkage groups were assigned to the 12 Chrs based on markers’ physical positions (McCouch *et al.* 2002). Orders of closely linked (a few cMs) markers were also checked for physical positions on the Nipponbare genome sequence (International Rice Genome Sequencing Project 2005).

### Data and QTL analysis

Germination data from the BC_1_F_1_ population were used to infer correlations for the degree of dormancy between seeds and caryopses and between the AR treatments. Data from the BC_2_F_1_ populations were used to estimate correlations of SD with each of the morphological traits. Linear correlation analysis was performed using the SAS CORR program.

For QTL analysis, germination data (*x*) were transformed by sin^−1^(*x*)^−0.5^ to improve the normality. The analysis was performed using Windows QTL Cartographer V2.5_011 (Wang *et al.* 2012). The interval mapping program was used to scan for QTL at 1 cM walking speed and 1000 permutations at 5% error rate. The composite interval mapping program was used to define QTL peak positions and C.I. (one-LOD support regions), and to estimate the effects of the mapped loci and their contributions to the phenotypic variances (*R*^2^).

### Data availability

Data for the origin and differentiation of the parental lines and data for trait correlations in the BC populations are available as Tables S1–S3 in File S1. Phenotypic and genotypic datasets from the mapping populations are available upon request.

## Results

### Genetic differentiation and linkage map

F_1_ plants from the EM93-1/LD cross had ∼65% seed set, which was ∼30% lower than the seed set rate for F_1_ EM93-1/SS18-2 plants (Gu *et al.* 2004). Partial sterility is a characteristic of hybrid F_1_s from an intersubspecific cross in rice (Oka 1988). The common parent EM93-1 in the two crosses is an *indica* line. The lower seed set rate in the EM93-1/LD cross supports that LD is *japonica*-like (Tang and Morishima 1997) and genetic differentiation between the parents is greater than the level for an introsubspecific cross.

**Figure 1:**
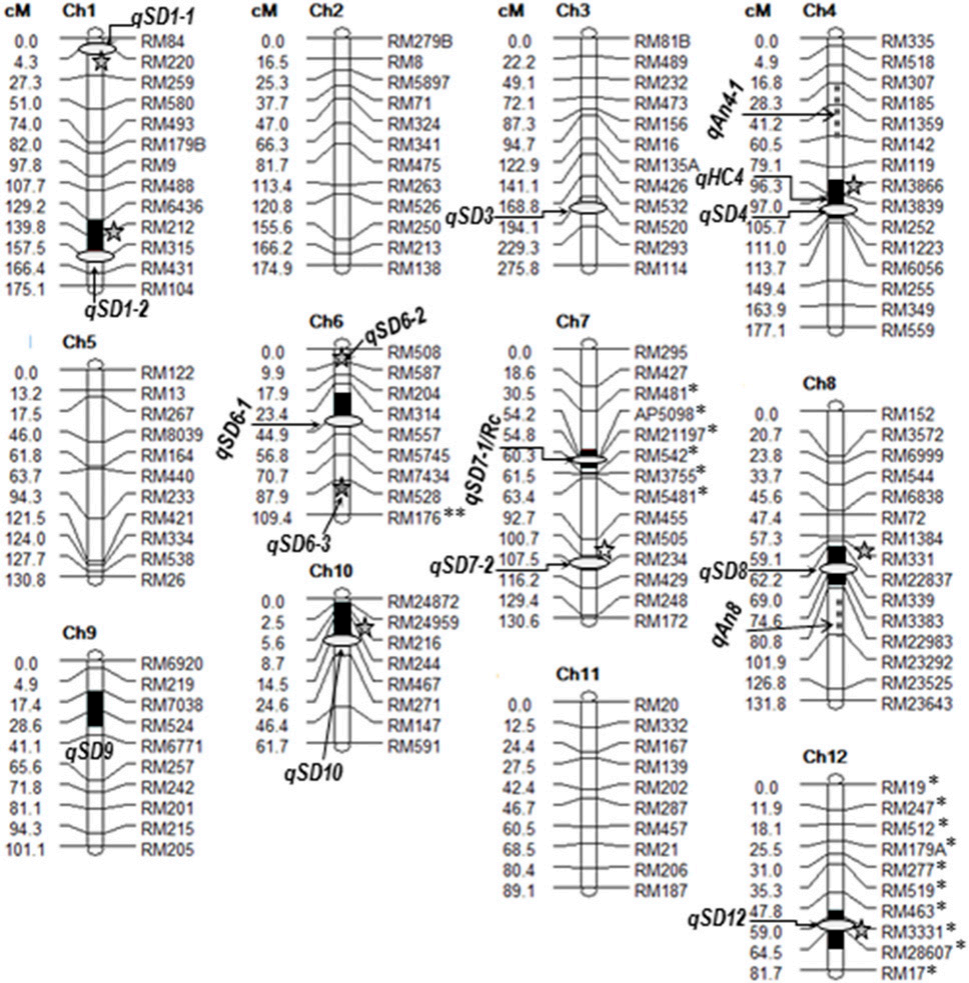
A framework linkage map and positions of seed dormancy QTL identified in this and previous research. The map was marked with RM loci segregating in the BC_1_F_1_ EM93-1//EM93-1/LD population. Asterisks indicate segregation distortion loci in favor of the allele from EM93-1 (*) or LD (**). Black bars indicate C.I. (equivalent to one-LOD support lengths) of qSD, qAn, or qHC detected in this BC_1_F_1_ population (Figure 2B). Five-point stars indicate qSDs detected in the BC_2_F_1_ (9) (Figure 3) and/or BC_2_F_1_ (139) (Figure 4), but not in this BC_1_F_1_ population. Ovals indicate approximate positions of qSDs previously detected in the BC_1_F_1_ EM93-1//EM93-1/SS18-2 population or its advanced generations (Gu *et al.* 2004; Ye *et al.* 2010). LD and SS18-2 are temperate and tropical ecotypes of weedy rice, respectively. Ch, chromosome; LD, pure line LüDao; LOD, logarithm of the odds; qAn, QTL for awn; qHC, QTL for hull color; qSD, QTL for seed dormancy; QTL, quantitative trait loci; RM, rice microsatellite.

DNA polymorphism between EM93-1 and LD was ∼60%, based on 256 markers amplified for alleles of predicted molecular sizes. Based on the BC_1_F_1_ EM93-1//EM93-1/LD (LD-BC_1_F_1_) population, a linkage map (Figure 1) was constructed using 139 markers, with 0.027% missing values. This map covered a total of 1650 cM for the 12 Chrs, with the mean intermarker distance being 12.9 (± 8.9) cM. About 45% of the 139 markers were also located on the linkage map constructed using the BC_1_F_1_ EM93-1//EM93-1/SS18-2 (SS18-BC_1_F_1_) population (Gu *et al.* 2004). The total genetic distance was 250 cM shorter for the LD than for the SS18-2 genome in the EM93-1 background.

Segregation distortion was observed for some or all of the markers on Chrs 6, 7, and 12 in the LD-BC_1_F_1_ population (Figure 1). The segregation ratio was biased against heterozygotes for the markers on Chrs 7 and 12, but toward the heterozygote for RM176 located near the end of the long arm of Chr 6 (Table 1). Because the BC_1_F_1_ population was developed using the F_1_ EM93-1/LD plants as the male parent, the distortions must be caused by functionally differentiated genes for male gametophyte development or pollination of the hybrid (sporophyte). Such sporo-gametophytic interaction genes were associated with partial sterility of hybrids from distant crosses in the *O. sativa* complex (Oka 1988). In contrast, a segregation distortion was not detected for any of the loci on Chrs 6 and 7, and the markers distal to the major SD QTL *qSD12*, in the SS18-BC_1_F_1_ population (Gu *et al.* 2004).

**Table 1:** Summary of segregation distortion loci linked to seed dormancy QTL in the BC_1_F_1_ (EM93-1//EM93-1/LD) and BC_2_F_1_ populations

Locus (QTL)	Chr	Population	Number of Plants	Genotypic (Allelic) Frequency		Chi-Square Value*^a^*
				Homozygote (Allele from EM93-1)	Heterozygote (Allele from LD)	
RM176 (*qSD6-3*)	6	BC_1_F_1_	163	0.31	0.69	6.09*
		BC_2_F_1_ (9)	136	0.25	0.75	8.50**
		BC_2_F_1_ (139)	149	0.09	0.91	24.57***
RM21197 (*qSD7-1*)	7	BC_1_F_1_	163	0.69	0.31	5.71*
		BC_2_F_1_ (9)	143	0.76	0.24	9.83**
		BC_2_F_1_ (139)	152	0.59	0.41	1.11*^b^*
RM28607 (*qSD12*)	12	BC_1_F_1_	163	0.86	0.14	21.00***
		BC_2_F_1_ (9)	143	0.94	0.06	28.20***

QTL, quantitative trait loci; Chr, chromosome; LD, pure line LüDao.

a Significance of the deviation from the 1:1 expectation at probability levels of * *P* < 0.05, ** *P* < 0.01, or *** *P* < 0.0001.

b This population was segregating for a short segment containing *qSD7-1/* RM21197 on Chr 7 (Figure 4A) that may not encompass the segregation distortion locus.

### QTL associated with SD in the BC_1_F_1_ population

**Figure 2:**
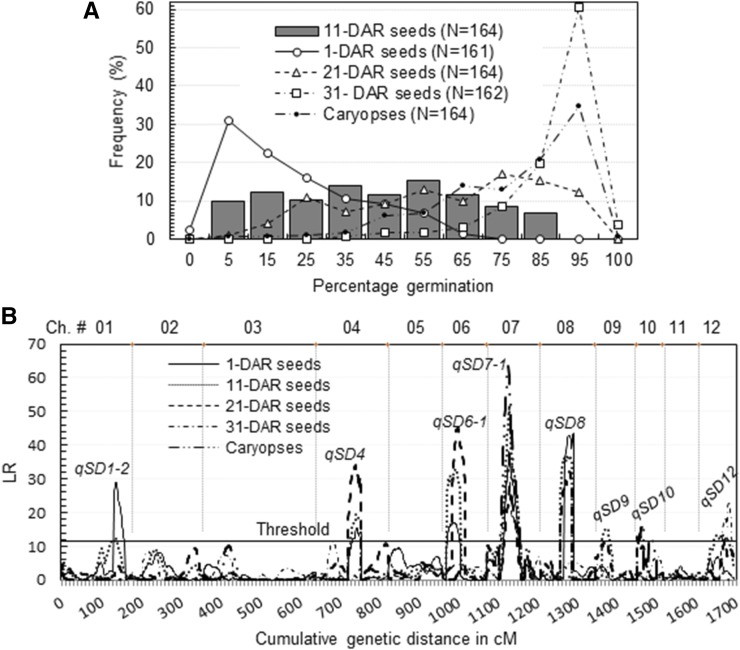
Genome-wide scan for seed dormancy QTL in the BC_1_F_1_ EM93-1//EM93-1/LD population. (A) Frequency distributions of percent germination for seeds or caryopses. *N* was the number of plants evaluated for germination at DAR. (B) Distributions of LR along the 12 Ch. Seed dormancy QTL (qSD) were inferred by peaks of the LR distributions above the threshold. Ch, chromosome; DAR, days after ripening; LR, likelihood ratio; qSD, QTL for seed dormancy; QTL, quantitative trait loci.

The frequency distribution pattern of percent germination varied with seeds, caryopses, or days of AR (DAR) in the LD-BC_1_F_1_ population (Figure 2A). Correlations (r) of seed germination between any two of the 1, 11, 21, and 31 DAR treatments were significant (Table S2 in File S1), but coefficients of determination were relatively low (*R*^2^ = 0.30–0.77). Similarly, the degree of dormancy between seeds and caryopses was positively correlated, but the *R*^2^ values (0.17–0.21) were even lower than the estimates for seeds at the different DAR (Table S2 in File S1). Therefore, all these measurements were used to detect qSD.

A total of eight qSDs were detected in the population (Figure 2B). Of them, *qSD7-1* was the only one whose effect could be detected by seeds at 1, 11, 21, and 31 DAR. This major QTL contributed more to the variance in germinability for caryopses (*R*^2^ = 0.27) than for seeds (*R*^2^ = 0.07–0.15), and was collocated with *Rc*. The collocation accounted for the phenotypic correlation between the dormancy and pericarp color traits (Gu *et al.* 2005a). The remaining loci were associated with one to three of the five measurements (Table 2). LD and EM93-1 contribute the dormancy-enhancing allele to seven and one (*qSD1-2*) of the eight QTL, respectively.

**Table 2:** Summary of QTL for seed dormancy (qSD) identified in the BC_1_F_1_ (EM93-1//EM93-1/LD) and BC_2_F_1_ populations

QTL	Chr	Peak (cM)*^a^*	LR*^b^*	*R*^2^*^b^*	Effect*^c^*	Germination*^c^*	Population
*qSD1-1*	1	RM220 (4)	38.1	0.18	−0.16	14 DAR	BC_2_F_1_ (139)
*qSD1-2*	1	RM212 (−2)	28.9	0.10	0.15	1 DAR	BC_1_F_1_
		RM212 (−2)	12.4	0.03	0.10	11 DAR	
*qSD4*	4	RM3839 (3)	15.2	0.05	−0.11	1 DAR	BC_1_F_1_
		RM3839 (2)	19.4	0.05	−0.13	11 DAR	
		RM3839 (1)	34.1	0.14	−0.23	21 DAR	
*qSD6-1*	6	RM314 (7)	17.0	0.06	−0.12	1 DAR	BC_1_F_1_
		RM314 (8)	32.7	0.11	−0.18	11 DAR	
		RM314 (14)	45.1	0.15	−0.23	21 DAR	
		RM557 (0)	11.6	0.05	−0.10	14 DAR	BC_2_F_1_ (139)
*qSD6-2*	6	RM587 (0)	14.9	0.07	−0.08	7 DAR	BC_2_F_1_ (139)
*qSD6-3*	6	RM528 (16)	11.3	0.08	−0.15	7 DAR	BC_2_F_1_ (9)
		RM528 (13)	19.3	0.16	−0.28	14 DAR	
		RM528 (−3)	27.1	0.18	−0.16	7 DAR	BC_2_F_1_ (139)
		RM528 (6)	33.6	0.13	−0.15	14 DAR	
*qSD7-1*	7	RM21197 (0)	38.0	0.12	−0.19	1 DAR	BC_1_F_1_
		RM21197 (0)	51.8	0.15	−0.24	11 DAR	
		RM21197 (1)	27.5	0.07	−0.18	21 DAR	
		RM21197 (0)	32.6	0.13	−0.17	31 DAR	
		RM21197 (−1)	63.3	0.27	−0.30	Caryopsis	
		RM21197 (0)	19.3	0.10	−0.19	7 DAR	BC_2_F_1_ (9)
		RM21197 (2)	15.1	0.10	−0.32	14 DAR	
		RM21197 (0)	14.8	0.07	−0.06	7 DAR	BC_2_F_1_ (139)
		RM21197 (−1)	16.1	0.08	−0.11	14 DAR	
*qSD7-2*	7	RM505 (1)	17.2	0.09	0.15	7 DAR	BC_2_F_1_ (9)
		RM505 (4)	13.1	0.09	0.27	14 DAR	
*qSD8*	8	RM339 (−1)	42.9	0.18	−0.14	1 DAR	BC_1_F_1_
		RM339 (−2)	36.8	0.10	−0.18	11 DAR	
		RM339 (−0)	31.9	0.09	−0.17	21 DAR	
		RM339 (−2)	12.1	0.06	−0.13	7 DAR	BC_2_F_1_ (9)
*qSD9*	9	RM524 (−1)	15.2	0.04	−0.11	11 DAR	BC_1_F_1_
		RM524 (−3)	11.5	0.03	−0.10	21 DAR	
*qSD10*	10	RM244 (−2)	12.5	0.07	−0.17	Caryopsis	BC_1_F_1_
		RM244 (3)	16.8	0.07	−0.10	14 DAR	BC_2_F1 (139)
*qSD12*	12	RM28607 (−2)	13.6	0.04	−0.14	11 DAR	BC_1_F_1_
		RM28607 (−4)	11.6	0.03	−0.13	21 DAR	
		RM28607 (3)	23.3	0.16	−0.23	31 DAR	
		RM28607 (5)	61.7	0.67	−1.07	21 DAR	BC_2_F_1_ (9)

**Figure 3:**
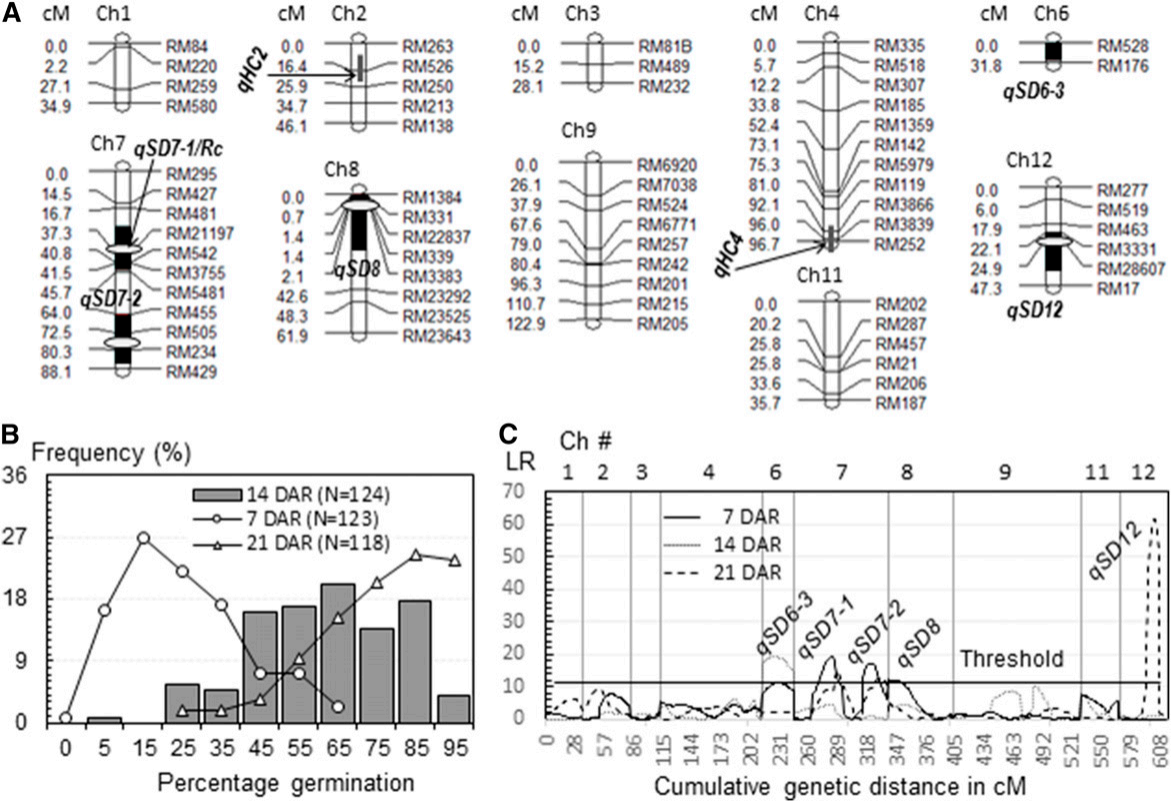
Mapping of seed dormancy QTL in the BC_2_F_1_ (9) population. (A) A partial linkage map. The map was constructed with markers on 10 Ch or Ch segments segregating in the population. Black bars indicate one-LOD support lengths for qSD, qAn, or qHC. Ovals indicate positions of qSDs previously detected in the BC_1_F_1_ EM93-1//EM93-1/SS18-2 population (Gu *et al.* 2004). (B) Frequency distributions of percent germination. N was the number of BC_2_F_1_ plants evaluated at 7, 14, or 21 DAR. (C) Distributions of LRs along the map. qSDs were inferred by peaks of the LR distributions above the threshold. Ch, chromosome; DAR, days of after-ripening; LOD, logarithm of the odds; LR, likelihood ratio; qAn, QTL for awn; qHC, QTL for hull color; qSD, QTL for seed dormancy; QTL, quantitative trait loci.

**Figure 4:**
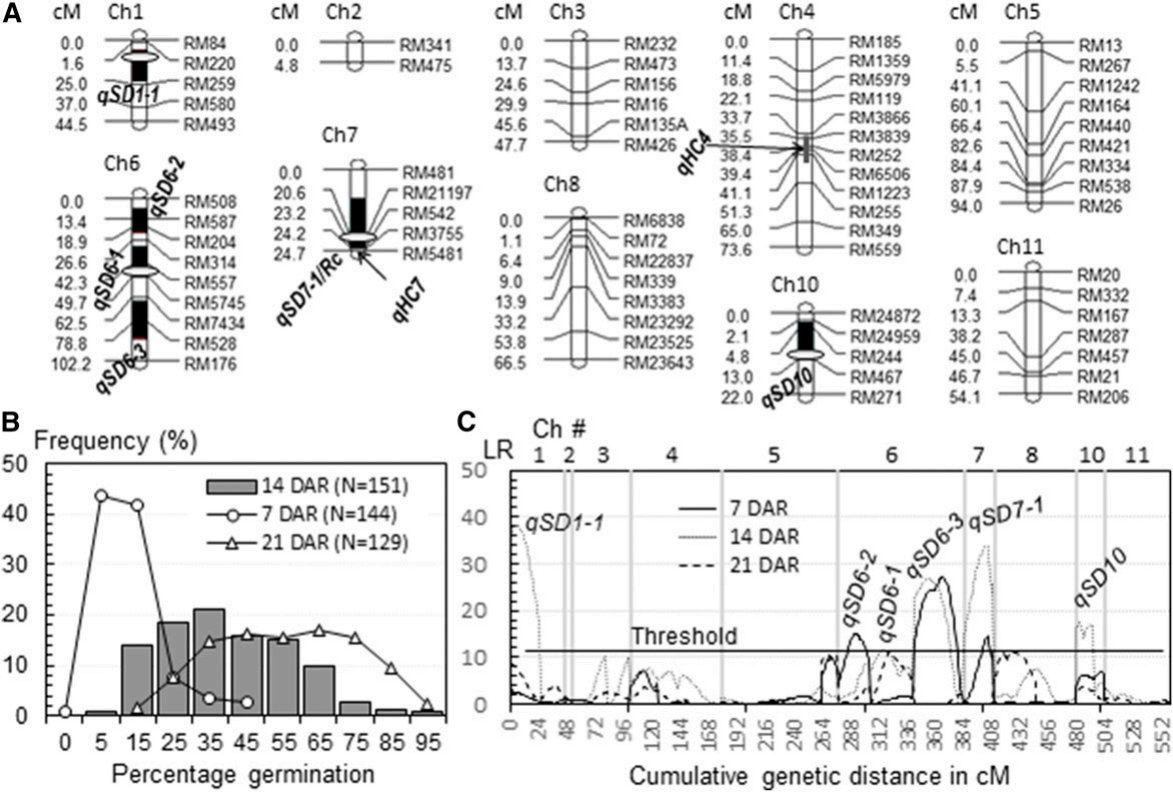
Mapping of qSD in the BC_2_F_1_ (139) population. (A) A partial linkage map. The map was constructed with markers on 10 Ch or Ch segments segregating in the population. Black bars indicate one-LOD support intervals for qSD, qAn, or qHC. Ovals indicate positions of qSDs previously detected in the BC_1_F_1_ EM93-1//EM93-1/SS18-2 population (Gu *et al.* 2004, 2005b; Ye *et al.* 2010). (B) Frequency distributions of percent germination. *N* was the number of BC_2_F_1_ plants evaluated at 7, 14, or 21 DAR. (C) Distributions of LRs along the map. qSDs were inferred by peaks of the LR distributions above the threshold. Ch, chromosome; DAR, days after ripening; LOD, logarithm of the odds; LR, likelihood ratio; qAn, QTL for awn; qHC, QTL for hull color; qSD, QTL for seed dormancy; QTL, quantitative trait loci.

QTL, quantitative trait loci; Chr, chromosome; LR, likelihood ratio; DAR, days after ripening; qSD, QTL for seed dormancy.

a Number in the parentheses is the genetic distance of the peak located above (−) or below the marker on the Chr or Chr segment in Figure 1, Figure 3A, or Figure 4A.

b LR and proportion of the variance explained by the QTL (*R*^2^).

c Difference between the heterozygous and homozygous genotypes at the locus in arcsine-transformed percent germination for intact seeds at DAR or for caryopsis.

### QTL associated with SD in the BC_2_F_1_ (9) population

BC_1_F_1_ plant #9 is heterozygous for Chr 9 and part of the others except Chrs 5 and 10, while the remainder of the plant genome was synchronized by EM93-1. The total length of the heterozygous regions on the 10 Chrs is ∼600 cM, as estimated based on the BC_2_F_1_ (9) population of 130 plants (Figure 3A). The heterozygous regions cover peak-containing (one-LOD support) intervals for *qSD7-1*, *8*, *9*, and *12* detected in the BC_1_F_1_ population. Phenotypic variation for SD at 7, 14, and 21 DAR (Figure 3B), and segregating distortion for the three loci (Table 1), were observed in the BC_2_F_1_ (9) population. A total of five qSDs, including *qSD7-1*, *8*, and *12*, but not *qSD9*, were detected in the advanced BC population (Figure 3C). The new locus *qSD6-3* was located on the RM528-176 interval near the end of the long arm of Chr 6, and has the dormancy-enhancing allele from LD (Table 3). The other new locus, *qSD7-2*, is the second QTL on Chr 7 and has the dormancy-enhancing allele from EM93-1.

**Table 3:** Summary of QTL for the awn and hull color traits identified in the BC_1_F_1_ (EM93-1//EM93-1/LD) and BC_2_F_1_ populations

QTL	Chr	Peak (cM)*^a^*	LR*^b^*	*R*^2^*^b^*	Effect*^c^*	Measurement*^d^*	Population
Awn							
* qAn4-1*	4	RM185 (−1)	36.8	0.12	22.2	% Awned seeds	BC_1_F_1_
		RM185 (3)	33.4	0.24	39.0	% Awned seeds	BC_2_F_1_ (9)
		RM185 (0)	43.8	0.25	4.7	Awn length	
		RM5979 (1)	26.5	0.11	26.1	% Awned seeds	BC_2_F_1_ (139)
		RM5979 (1)	32.2	0.15	6.3	Awn length	
* qAn8*	8	RM23292 (0)	70.1	0.25	29.9	% Awned seeds	BC_1_F_1_
		RM23292 (−17)	125.3	0.59	74.5	% Awned seeds	BC_2_F_1_ (9)
		RM23292 (−19)	26.5	0.18	3.8	Awn length	
		RM23292 (−8)	57.9	0.33	47.9	% Awned seeds	BC_2_F_1_ (139)
		RM23292 (−1)	17.8	0.07	5.2	Awn length	
Hull color							
* qHC2*	2	RM526 (3)	13.5	0.05	−4.2	L* (Blackness)	BC_2_F_1_ (9)
			16.6	0.08	−0.7	a* (Redness)	
* qHC4*	4	RM252 (0)	126.7	0.54	0.7	Visual score	BC_1_F_1_
		RM252 (0)	39.1	0.17	−8.8	L* (Blackness)	BC_2_F_1_ (9)
		RM252 (0)	59.3	0.33	−1.3	a* (Redness)	
		RM252 (0)	26.4	0.15	−18	L* (Blackness)	BC_2_F_1_ (139)
		RM252 (0)	34.0	0.17	−1.3	a* (Redness)	
		RM252 (0)	37.0	0.20	−6.2	b* (Yellowness)	
* qHC7*	7	RM5481 (1)	15.7	0.08	0.7	a* (Redness)	

QTL, quantitative trait loci; Chr, chromosome; LR, likelihood ratio.

a Number in the parentheses is the genetic distance of the peak located above (−) or below the marker on the Chr or Chr segment in Figure 1, Figure 3A, or Figure 4A.

b LR and proportion of the variance explained by the QTL (*R*^2^).

c Difference between the heterozygous and homozygous genotypes in the trait value.

d The trait awn was measured by the percentage of seeds with awn and the mean awn length for seeds from a plant; and the hull color was measured by visual scores (dark *vs.* straw) for the BC_1_F_1_ population and by reflection spectrum readings for darkness (L*), red redness (a*), and yellowness (b*) for the BC_2_F_1_ population.

*qSD12* accounted for 67% of the variance in germination percentage at 21 DAR when effects of the other QTL were not significant in the BC_2_F_1_ (9) population (Table 2). However, *qSD12*’s effect was not significant at 7 and 14 DAR when the others were detectable. These results suggest that *qSD12* maintained an inhibitory effect on germination longer than the other SD QTL. In addition, the severe segregation distortion for the *qSD12*-containing region, which greatly reduced the genotypic frequency for heterozygotes (6%) in the BC_2_F_1_ population (Table 1), must also lower the power to evaluate the QTL’s effect on germination at an early stage of AR.

### QTL associated with SD in the BC_2_F_1_ (139) population

The BC_1_F_1_ plant #139 is heterozygous for Chr 6 and part of the others except Chrs 9 and 12, while the remainder of the plant genome was synchronized by EM93-1. The total length of the heterozygous regions on the 10 Chrs is ∼550 cM, as estimated based on the BC_2_F_1_ (139) population of 151 plants (Figure 4A). The heterozygous regions cover peak-containing intervals of *qSD4*, *6-1*, *7-1*, *8*, and *10* detected in the BC_1_F_1_, and *qSD6-3* detected in the BC_2_F_1_ (9) population. Phenotypic variation for SD at 7, 14, and 21 DAR (Figure 4B), and segregation distortion for RM176 on Chr 6 (Table 1), were observed in this population. A total of six qSDs, including *qSD6-1*, *6-3*, *7-1*, and *10* were detected (Figure 4C), but *qSD4* and *8* were not significant in the advanced BC population. Two new loci (*qSD1-1* and *6-2*) were identified in the BC_2_F_1_ population and both have the dormancy-enhancing allele from LD (Table 2). Of the three QTL on Chr 6, *qSD6-3* contributed most to the phenotypic variance (Table 2).

The BC_2_F_1_ (139) population segregated on Chr 7 for a segment of ∼30 cM encompassing *qSD7-1* and its flanking markers from RM481 to RM5481 (Figure 4A). However, segregation distortion was not detected for these markers, including RM21197 located within the *qSD7-1* underlying gene (Gu *et al.* 2011). This result indicates that the gene responsible for the segregation distortion in the BC_1_F_1_ and BC_2_F_1_ (9) populations locates outside the 30 cM segment.

### QTL associated with the seed morphological traits

Phenotypic variation for each of the morphological traits and their correlations with SD were observed in the two BC_2_F_1_ populations, with the presence of awn, dark pigment on the hull, or red pigment on the pericarp tissue tending to reduce germination percentage (Table S3 in File S1). The phenotypic correlations were similar to those observed in the three BC_1_F_1_ populations derived from different lines of weedy rice, including LD and SS18-2 (Gu *et al.* 2005a). Two awn QTL (*qAn4-1* and *8*) were detected in each of the LD-BC_1_F_1_ and two BC_2_F_1_ populations (Table 3). In the BC_2_F_1_ populations, the contribution of *qAn8* to the phenotypic variance (*R*^2^) was three to four times greater for the percentage of awned seeds than for awn length, while *qAn4-1* contributed almost equally to the two measurements. *qAn4-1* and *8* were linked to but not collocated with *qSD4* and *8*, respectively (Figure 1).

A major QTL (*qHC4*) and two modifiers (*qHC 2* and *7*) were associated with hull color (Table 3). *qHC4* was detected in all of the three populations and contributed most to phenotypic variances in the visual score and component reflection spectra. This major QTL was collocated with *qSD4* (Figure 1). The modifiers *qHC2* and *7* were detected in the BC_2_F_1_ #9 and #139 populations, respectively. *qHC7* contributed 8% to the phenotypic variance for the red reflectance only and was collocated with *qSD7-1/Rc* (Figure 4A). It is likely that the modifier *qHC7* could be the *Rc* locus, which was associated with the visual score for the pericarp color in the two BC_2_F_1_ populations. This is because the red pigment can be seen on intact straw hull-colored seeds.

## Discussion

### Similarity of SD genes between distinct ecotypes of weedy rice

A total of 12 SD QTL were identified from the primary and advanced BC populations developed using LD as the nonrecurrent parent. Two-thirds (eight) of these loci were detected from the BC_1_F_1_ population, and the remaining 1/3 identified from the BC_2_F_1_ populations where ∼65% of the genome was synchronized by the recurrent parent EM93-1. The temperate ecotype line LD has dormancy-enhancing alleles at 10 (83%) of the 12 loci. The estimate of 83% is close to the previous observation that the tropical ecotype line SS18-2 has dormancy-enhancing alleles at 80% of the 10 QTL detected in the EM93-1 background (Ye *et al.* 2010).

The SD QTL identified from the populations with LD or SS18-2 as the nonrecurrent parent represent a majority of the reported loci differentiated between wild/weedy and cultivated rice in regard to approximate map positions. For example, *qSD6-1*, *2*, and *3* are similar to those on Chr 6 reported for wild (Cai and Morishima 2000) and weedy (Jing *et al.* 2008) rice; *qSD4*, *7-1*, and *7-2* were located on the same marker intervals as the three QTL reported for the three accessions of weedy rice from USA (Subudhi *et al.* 2012; Mispan *et al.* 2013); and *qSD1-1* (Figure 1A) and *sd1* reported for wild rice (Li *et al.* 2006) were both mapped on the top of Chr 1. However, some loci reported by the other groups, such as *qSD-2* (Jing *et al.* 2008), *qSD3* (Subudhi *et al.* 2012), and *sd12* (Li *et al.* 2006), were not detected our research. It is possible that some SD genes could have been eliminated from founders of the LD and SS18 populations, or lost during evolution of the weed ecotypes.

The SS18 (∼7°N) and LD (∼34°N) populations acquired a similar level of SD in geographically isolated ecosystems because they share most genes for the adaptive trait. There are nine common loci (*qSD1-1*, *1-2*, *4*, *6-1*, *7-1*, *7-2*, *8*, *10*, and *12*) that are functionally differentiated for SD in both EM93-1/SS18-2 and EM93-1/LD crosses. It is estimated that the tropical and temperate ecotypes are similar in genotype for 75% of the 12 SD loci, if multiple alleles (more than two at a locus) are ignored. The estimated degree of similarity is similar to the report for the dicot model *Arabidopsis thaliana* (Bentsink *et al.* 2010). For example, of a total of 11 SD QTL identified for six ecotypes in the Landsberg *erecta* background, nine (82%) had an effect on delay of germination in two or more of the *Arabidopsis* populations (Bentsink *et al.* 2010). Thus, the comparative mapping results from the monocot and dicot models strongly suggest that naturally occurring genes controlling SD in a species were highly conserved during evolution.

### Evolutionary mechanisms of SD

The adaptive significance of SD relies on functionally differentiated alleles at multiple loci to regulate the time of germination in local ecosystems. This and the previous research in weedy rice revealed several mechanisms involved in regulating genotypic/allelic frequencies at SD loci in both natural and agricultural ecosystems. The first mechanism is direct selection for the time of germination, which is critical for locally adapted genotypes to complete their life cycle. An extreme example is the domestication of cereal crops by artificial selection for rapid germination mutants (Harlan 1965). The second mechanism is indirect selection for wild-type characteristics correlated with SD. A phenotypic selection for the presence of awn, dark hull, or red pericarp tended to enhance SD (Table S3 in File S1). The indirect selections retained the dormancy-enhancing alleles at the loci (*e.g.*, *qSD4*, *7-1*, and *8*) linked to or collocated with the genes for the interrelated traits (Figure 1; Gu *et al.* 2005b; Mispan *et al.* 2013). Some of the “linkage drags” or collocations could be pleiotropic effects of single genes. For example, *qSD7-1* and *Rc* are underlain by the same transcription factor gene (*Os07g1120*) regulating both abscisic acid (a dormancy-inducing hormone) and flavonoid (red pigments) biosynthesis pathways in early developing seeds (Gu *et al.* 2011). The indirect selections explain why SD is generally stronger in black-hulled awned “red” rice populations than in those without the wild-type characteristics (Delouche *et al.* 2007). Genome-wide phylogenetic analyses revealed that the hull color, pericarp color, and awn gene-containing regions were intensively selected during domestication and are informative for research on origins of weedy rice (Qiu *et al.* 2014; Kanapeckas *et al.* 2016; Li *et al.* 2017).

The third mechanism is indirect selection for crop-mimic traits, such as plant height and flowering time. LD contains dormancy-reducing alleles at *qSD1-2* and *qSD7-2*. Both loci also have an effect on plant height, when the QTL alleles were introduced from SS18-2 into the EM93-1 background (Ye *et al.* 2013). *qSD1-2* was cloned as *semidwarf1* (*sd1*), a major gene for plant height. The semidwarf line EM93-1 carries a dormancy-enhancing allele, while a vast majority of wild/weedy rice lines (including LD and SS18-2) have a dormancy-reducing allele at *qSD1-2*/*sd1* (Ye *et al.* 2015). A high frequency of the dormancy-reducing allele in the nondomesticated germplasm is indicative that the natural selection on such a pleiotropic gene has a greater impact on plant height than on dormancy. Collocation was also reported for the SD/heading date QTL on Chr 3 (*Sdr1/Hd8*; Takeuchi *et al.* 2003) and 6 (*qSD-6-2/qHD-6*; Jing *et al.* 2008), with the dormancy-enhancing alleles delaying flowering. Thus, correlational selections for crop-mimic traits may not favor the retention of a dormancy-enhancing allele, but could contribute to genetic diversity in germination capacity.

The other mechanism was inferred by the three segregation distortion loci (SDL) linked to (*qSD7-1*) or collocated with (*qSD6-3* and *12*) an SD locus. The segregation distortion favored a transmission of the dormancy-reducing alleles at *qSD7-1* and *12*, or the dormancy-enhancing allele at *qSD6-3*, through gametes produced by heterozygotes from the EM93-1/LD cross (Table 1). A similar pattern of segregation distortion was also observed for a *qSD12*-containing region, when it was heterozygous for the alleles from SS18-2 and EM93-1; this SDL had a larger effect on eliminating the dormancy-enhancing allele through the male than through the female gametes (Gu *et al.* 2015). Natural hybridization occurs between weedy and cultivated rice at a low rate (Oka 1988; Delouche *et al.* 2007). Thus, gametophyte development genes that cause segregation distortions in hybrid populations could also influence allelic frequencies for some SD loci, but the influence varies depending on crosses.

### Possible applications of SD genes

In addition to understanding the origins of conspecific/congeneric weeds in agroecosystems (Qiu *et al.* 2014; Kanapeckas *et al.* 2016; Li *et al.* 2017), SD genes conserved across ecotypes could be manipulated to develop a TM strategy. The TM strategy was proposed to complement transgene containment techniques to reduce the risk of gene flow from genetically modified (GM) crops to wild relatives (Gressel 1999). The basic concept is a built-in linkage between a fitness-enhancing transgene (*e.g.*, herbicide resistance) and a mitigating factor (*e.g.*, reduced SD), which has no negative effect on the GM crop but could reduce the adaptability of weed/crop hybrids to lower the transgene’s frequency in a weed population across generations (Gressel 1999). Silencing SD genes could promote germination uniformity (as for cereal cultivars) and make weeds relatively easy to eliminate by agronomic practices. We are using the *qSD7-1* and *12* underlying genes as silencing targets and the RNA interference and genome editing techniques to prove the TM concept in weedy red rice.

## Supplementary Material

Supplemental material is available online at www.g3journal.org/lookup/suppl/doi:10.1534/g3.117.040451/-/DC1.
