# Supplementary material for: Comparative Mapping of Seed Dormancy Loci Between Tropical and Temperate Ecotypes of Weedy Rice (Oryza sativa L.)
Source: G3 (Bethesda). 2017 Jun 6;7(8):2605–14. doi: 10.1534/g3.117.040451 (PMC5555466; doi:10.1534/g3.117.040451)
Supplement: Supplementary file 1 [file 2605File001.pdf]

Comparative Mapping of Seed Dormancy Loci between Tropical and Temperate Ecotypes of Weedy Rice (*Oryza sativa* L.)

Supplemental Material

Table S1 List of the rice lines (*O. sativa*) used as the parents for hybridization

Line	Origin	Ecotype or subspecies	Hull color	Pericarp color	Awn	Seed dormancy
LD (C9587*)	Lianyungang, China 34.59°N 119.22°E**	Temperate weedy, *japonica*-like*	Black	Red	Long (>5 cm)	Strong
SS18-2 (SS18*)	Songkla, Thailand 7.18°N 100.59°E*	Tropical weedy, *indica*-like*	Black	Red	Long (>5 cm)	Strong
EM93-1	Breeding line	*indica*, cultivated	Straw	White	None	Weak

Note: EM93-1 was the common parent in crosses with LD or SS18-2. Superscripts indicate the information from Tang and Morishima (1997) (*) or Jiang *et al*. (1985) (**).

Table S2 List of correlations between seeds and caryopses for the degree of dormancy in the BC_1F_1 (EM93-1//EM93-1/LD) population

Measurement	11-DAR seeds	21-DAR seeds	31-DAR seeds	Caryopses
1-DAR seeds	0.878	0.742	0.546	0.406
11-DAR seeds		0.871	0.652	0.414
21-DAR seeds			0.724	0.454
31-DAR seeds				0.414

Listed are Pearson correlation coefficients (r) of percent germination for seeds at 1 to 31 days of after-ripening (DAR) or caryopses at 1 DAR. All the *r* values were significant at the probability level of <0.0001.

Table S3 Phenotypic variation in seed morphologies and their correlations with seed dormancy in the BC_2F_1 (9) and BC_2F_1 (139) populations

Seed dormancy[a]	Parameter	Morphological traits[b]					
		Awn		Hull color			Pericarp color
		Length (mm)	%	L*	a*	b*	
BC_2F_1 (9)		(2.7±4.8)	(28±38)	(47±8.8)	(2.7±1.1)	(13.5±6.1)	(0.53±0.50)
7 DAR	*r*	-0.381	-0.408	0.420	0.528	0.486	-0.450
	Prob.	<.0001	<.0001	<.0001	<.0001	<.0001	<.0001
14 DAR	*r*	-0.426	-0.486	0.471	0.568	0.498	-0.413
	Prob.	<.0001	<.0001	<.0001	<.0001	<.0001	<.0001
21 DAR	*r*	-0.436	-0.496	0.451	0.498	0.468	-0.435
	Prob.	<.0001	<.0001	<.0001	<.0001	<.0001	<.0001
BC_2F_1 (139)		(7.1±8.0)	(50±38)	(45±9.0)	(2.4±1.1)	(13.3±6.0)	(0.63±0.48)
7 DAR	*r*	-0.181	-0.238	0.075	0.045	0.061	-0.321
	Prob.	0.0332	0.0048	0.3775	0.5998	0.4749	<.0001
14 DAR	*r*	-0.352	-0.392	0.275	0.241	0.252	-0.353
	Prob.	<.0001	<.0001	0.0010	0.0041	0.0026	<.0001
21 DAR	*r*	-0.346	-0.383	0.275	0.325	0.294	-0.376
	Prob.	<.0001	<.0001	0.0016	0.0002	0.0007	<.0001

[a] DAR, days of after-ripening prior to germination.

[b] Data in the parentheses are means and standard deviations for the trait measurements described in Materials and Methods. For the awn and pericarp color traits, a negative correlation coefficient (*r*) indicates that the presence of awn or red pigment tended to reduce germination (or enhance seed dormancy). For the hull color, a positive *r* value indicates that a high intensity of darkness (L*), greenness (a*) or blueness (b*) tended to reduce germination.
